# Overexpression of Cystatin SN positively affects survival of patients with surgically resected esophageal squamous cell carcinoma

**DOI:** 10.1186/1471-2482-13-15

**Published:** 2013-05-28

**Authors:** You-Fang Chen, Gang Ma, Xun Cao, Rong-Zhen Luo, Li-Ru He, Jie-Hua He, Zhi-Liang Huang, Mu-Sheng Zeng, Zhe-Sheng Wen

**Affiliations:** 1 State Key Laboratory of Oncology in South China, Cancer Center, Sun Yet-Sen University, No.651, Dongfeng Road East, Guangzhou, China; 2 Department of Thoracic Oncology, Cancer Center, Sun Yet-Sen University, No.651, Dongfeng Road East, Guangzhou, China; 3 Department of Critical Care Medicine, Cancer Center, Sun Yet-Sen University, No.651, Dongfeng Road East, Guangzhou, China; 4 Department of Pathology, Cancer Center, Sun Yet-Sen University, No.651, Dongfeng Road East, Guangzhou, China; 5 Department of Radiation Oncology, Cancer Center, Sun Yet-Sen University, No.651, Dongfeng Road East, Guangzhou, China

**Keywords:** Esophageal squamous cell carcinoma, Cystatin SN, Immunohistochemistry, Prognosis

## Abstract

**Background:**

Cystatin SN is a secreted protein and a cysteine proteinase inhibitor. It has been considered to be a tumor marker for gastrointestinal tract cancer in several functional researches. However, the clinicopathological and prognostic significance of Cystatin SN expression in esophageal squamous cell carcinoma (ESCC) has not been elucidated.

**Methods:**

In our study, the expression of Cystatin SN was detected in 209 surgically resected ESCC tissues and 170 peritumoral normal esophageal mucosae by immunohistochemistry. The prognostic significance of Cystatin SN expression was analysed with Kaplan-Meier plots and the Cox proportional hazards regression models.

**Results:**

The results showed that the immunostaining of Cystatin SN in ESCC tissues was less intense than that in the normal control tissue (*P* < 0.001). Compared with patients with low tumoral Cystatin SN expression, ESCC patients with tumors high-expression Cystatin SN exhibited increased disease-free survival (DFS) and overall survival (OS) (*P* < 0.001 and *P* < 0.001, respectively). Furthermore, the expression level of Cystatin SN could further stratify the ESCC patients by survival (DFS and OS) in the stage II subgroup (*P* < 0.001 and *P* < 0.001, respectively). Multivariate analyses showed that Cystatin SN expression, N status and differentiation were independent and significant predictors of survival.

**Conclusions:**

We concluded that ESCC patients whose tumors express high levels of Cystatin SN have favourable survival compared with those patients with low Cystatin SN expression. Tumoral Cystatin SN expression may be an independent predictor of survival for patients with resectable ESCCs.

## Background

Esophageal cancer (EC) is the fourth leading cause of cancer-related death worldwide, and there were approximately 482,300 new esophageal cancer cases and 406,800 EC-related deaths in 2008, with incidence rates varying almost 16-fold throughout the world [1]. China accounts for approximately 53.6% of new cases and 51.7% of EC-related worldwide [2]. In China, approximately 90% of all esophageal cancers are squamous cell carcinomas [2,3]. The best option for curing esophageal squamous cell carcinoma (ESCC) is surgical resection, but the delayed clinical presentation of symptoms (e.g., dysphagia and odynophagia) may result in the loss of the opportunity to undergo surgery. Even with the development of surgical techniques and better postoperative management, the 5-year survival rate of patients after complete surgical resection only ranges from 10% to 40% [4].

Cysteine proteases are involved in tissue remodeling during development, and they induce the migration of cancer cells [5,6]. The expression, function and location of many proteases are associated with tumor progression [5,7,8]. In the past, researchers have focused their attentions on tumor cells, but efforts have shifted to the role of cathepsins in the progression of tumor cells, with the goal of designing a novel protease-based drug to attenuate the invasive and metastatic capabilities of tumor cells [5]. Simultaneously, recent evidence suggests that lysosomal cysteine proteases play an important role in ESCC growth, invasion and metastasis [7,8].

The *CST1* gene encodes a secretory peptide called Cystatin SN, which is a cysteine proteinase inhibitor [9]. The balance between cathepsins and their cystatins has been reported to influence various pathological processes, including tumor invasion and metastasis [10,11]. Cystatin SN is one of the family 2 cystatins (including cystatins C, D, S, SA, SN, M and F), which are encoded by one subfamily of the *Cystatin* (*CST*) superfamily [12]. There have been reported that most of the family 2 Cystatins (excluding Cystatin SA) are closely associated with tumor metastasis and invasion [6,11,13-21]. In particular, CST1 plays an important role in the regulation of proteolysis and is highly involved in gastric tumorigenesis though TCF-mediated proliferative signalling [6]. At the same time, CST1 was also identified as a tumor marker for colorectal cancer [21] although this finding lacks the support of clinicalpathological data. All previous observations have implied that Cystatin SN may contribute to the process of carcinogenesis and tumor progression. However, the clinicopathological and prognostic significance of Cystatin SN in human ESCC has not yet been elucidated.

Based on these considerations, in our study, we analysed Cystatin SN protein expression in surgically resected ESCCs from a large patient cohort. Furthermore, we have discussed the clinicopathological and prognostic value of Cystatin SN expression in ESCCs.

## Methods

Between October 2000 and April 2007, 240 primary ESCC patients underwent complete surgical resection (R0) at the Sun Yat-sen University Cancer Center were eligible for our study. After exclusion of the noninformative samples (e.g., unrepresentative and lost samples), a total of 209 ESCC tissues and 170 peritumoral tissues were included for immunohistochemical analysis. All patients underwent a pretreatment evaluation (e.g., basic personal information, a complete history, a physical examination and a preoperative examination) and provided a complete follow-up data. The data regarding the tumor (e.g., tumor location, differentiation, T stage, N stage, distant metastasis) were collected from the postoperative pathological results and the preoperative examination. The tumor differentiation grades were based on the World Health Organization criteria, and the tumor-node-metastasis classifications were defined according to the the American Joint Committee on Cancer (AJCC, 2009). The study was approved by the Medical Ethics Committee of the Cancer Center at Sun Yat-Sen University.

Follow-up data after surgery (e.g., recurrence, metastasis, vital status, death and the causes of death) were obtained from the patients’ records. All patients were followed up every 4-6 months during the first 3 years and every year thereafter. All patients’ vital statuses were confirmed in January 2012.

### Immunohistochemistry (IHC)

A total of 209 ESCC tissues and 170 paired peritumoral normal esophageal tissues were fixed in 10% neutral buffered formalin after complete surgical resection (R0) at our cancer centre before being embedded in paraffin and used for pathological evaluation. All paraffin-embedded specimens used in this study were cut into 4 μm sections and baked for 1 h at 65°C. IHC staining was performed using the Dako Envision system (Dako, Carpinteria, CA) following the manufacturer’s recommended protocols, which have been described previously [22]. Briefly, all sections were deparaffinised and rehydrated and endogenous peroxidase activity was blocked prior to immunostaining. Then, the slides were processed for antigen retrieval by boiling in 10 mM citrate buffer (pH 6.0) for 5 minutes. After natural cooling, all sections were treated with a rabbit polyclonal cystatin SN antibody (1:800 dilution; NBP1-55,995, Novus, Littleton, USA) overnight at 4°C. Subsequently, the sections were incubated with a biotinylated secondary antibody for 30 min at 37°C. Finally, the sections were incubated with streptavidin-horseradish peroxidase complex and developed with diaminobenzidine (DAB). Mayer’s haematoxylin was used as a counterstain. For a negative control, the antibody was replaced with normal rabbit serum.

### IHC evaluation

The grading of the cytoplasmic Cystatin SN staining was performed using light microscopy to generate an immunoreactivity score (IRS) [23,24]. The IRS of CST1 was calculated by multiplying the intensity and extent scores. Entire sections were observed to assign the scores. The staining intensity was scored as 0 (no staining), 1 (weak staining, yellow brown), 2 (moderate staining, yellow brown), or 3 (strong staining, brown), and the percentage of positively stained cells was evaluated as 0 (0%), 1 (1% to 10%), 2 (11% to 50%), 3 (51% to 70%), or 4 (71% to 100%). In our study, we used the median of all IRSs (4.0) as the cut-off point [25]. The IRS was classified as: – (0 score, absent); 1+ (range from 1 to 4, weak); 2+ (range from 5 to 8 score, moderate); or 3+ (range from 9 to 12, strong). We defined – to 1+ as “Cystatin SN- low expression” and 2+ to 3+ as “Cystatin SN- high expression”.

The stained tissue sections were assessed and scored independently by two senior pathologists (Ruo-Zhen Luo and Jie-Hua He), both of whom were blinded to the clinical characteristics of the patients. The final score for Cystatin SN was defined using the average values of the two observers’ scores. To ensure the consistency of the scores, discordant cases were reviewed.

### Statistical analysis

All statistical analyses were performed using the SPSS 13.0 statistical software package (SPSS, Chicago, IL). The differences in the IRSs between ESCC and peritumoral normal esophageal tissues were calculated using the paired-sample Student test (paired-sample *t*-test). The Chi-squared test (χ^*2*^ test) was used to analyse the correlations between Cystatin SN expression and the clinicopathological characteristics of the ESCCs. Survival curves were plotted using Kaplan-Meier plots and Log-rank tests. Multivariate analysis was performed using the Cox proportional hazard method, which was performed for all significant variables using univariate analysis. Overall survival (OS) and disease-free survival (DFS) were calculated from the date of surgery to the date of death or the last follow-up and to the date of recurrence or distant metastasis, respectively. *P* < 0.05 was considered statistically significant.

## Results

### Patient characteristics

The clinicopathological characteristics of the 209 ESCC patients are summarised in Table 1. Up to the last follow-up visit (January 2012), the 1-, 3-, 5-, and 10-year survival rates for the whole cohort of patients were 83.7%, 56.9%, 50.1% and 0.96%, respectively. None patients received neoadjuvant treatment. Excluding the patients who had KPS < 70 or/and refused chemotherapy, 79 patients have completed systemic adjuvant chemotherapy (cisplatin-based combinations) (OS, 66 months; 5-years survival, 53.8%) after curative-intent surgery.

**Table 1:** Association between Cystatin SN expression and clinicopathological variables in 209 ESCC patients

**Variables**	**Cases**	**Cystatin SN expression (%)**		
		**Low (−to+)**	**High (++to+++)**	P value^*^
		**Number. (%)**	**Number. (%)**	
Age (years)				0.166
Median^†^	57			
Range	32-80			
≤ 57	113	83(73.5)	30(26.5)	
> 57	96	62(64.6)	24(35.4)	
Gender				0.328
Male	150	107(71.3)	43(28.7)	
Female	59	38(64.4)	21(35.6)	
Tumor location				0.699
Upper	15	9(60.0)	6(40.0)	
Middle	96	68(70.8)	28(29.2)	
Lower	98	68(69.4)	30(30.6)	
Differentiation				0.200
G1	56	39(69.6)	17(30.4)	
G2	105	68(64.8)	37(35.2)	
G3	48	38(79.2)	10(20.8)	
pT status				0.133
pT1	4	1(25.0)	3(75.0)	
pT2	62	42(67.7)	20(32.3)	
pT3	143	102(71.3)	41(28.7)	
pN status				0.176
pN0	116	76(65.5)	40(34.5)	
pN1-3	93	69(74.2)	24(25.8)	
pTNM status				0.183
II	133	88(66.2)	45(33.8)	
III	76	57(75.0)	19(25.0)	

^*^Chi-square test; ^†^median age was 57 years for 209 enrolled ESCC patients.

*G* grade, *pT* pathologic tumor, *pN* pathologic node, *pTNM* pathologic tumor-node-metastasis.

### Cystatin SN expression and its correlations with clinicopathological characteristics

**Figure 1:**
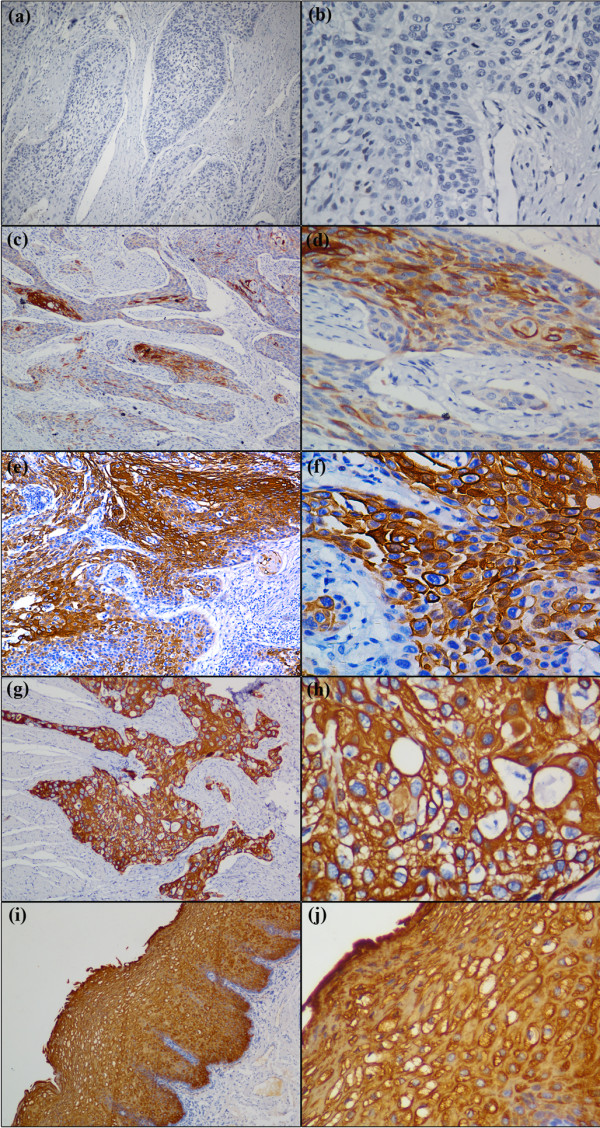
**Immunohistochemical staining of esophageal squamous cell carcinoma (ESCC) and peritumoral normal esophageal mucosae with anti-Cystatin SN.** Low expression of Cystatin SN were detected in ESCC tissues (**a**, **b** and **c**, **d**), and the IRS grades of (**a**, **b**) and (**c**, **d**) belong to absent(−) and weak(1+), respectively, in which (**a**, **c**) original magnification is × 100, and (**b**, **d**) original magnification is × 400, respectively; High expression of Cystatin SN was detected in ESCC tissues (**e**, **f** and **g**, **h**) and peritumoral normal esophageal mucosae( **i**, **j** ), and the IRS grades of (**e**, **f**) and (**g**, **h** and **i**, **j**) belong to moderate (2+) and strong (3+), respectively, in which (**e**, **g**, **i**) original magnification is × 100, and (**f**, **h**, **j**) original magnification is × 400, respectively.

Cystatin SN expression was observed predominantly in the cytoplasm of the tumor cells and normal squamous epithelial cells (Figure 1). Using the criteria described above, strong staining (3+) of Cystatin SN was detected in all of normal squamous epithelial cells, whereas various staining patterns were obtained in the ESCC tissues. There was a statistically significant difference between the Cystatin SN staining of the tumors and the normal tissues (*P* < 0.001, Table 2). However, there was no significant associations between the Cystatin SN expression of the ESCCs and age, gender, tumor location, differentiation, T status, N status, and pathological stage in the Chi-squared test (*P* > 0.05, Table 1).

**Table 2:** Immunohistochemistry results for Cystatin SN in esophageal squamous cell carcinoma (ESCC) compared with peritumoral normal esophageal tissues

**Cyatatin SN**	**ESCC (N = 209)**	**Peritumoral normal tissues (N = 170)**
0	29 (13.9%)	0
1+	116 (55.5%)	0
2+	28 (13.4%)	0
3+	36 (17.2%)	170 (100%)

### Correlations between Cystatin SN expression, the patients clinicopathological characteristics and survival

Up to the last follow-up date, 119 cancer-related ESCC deaths were observed. The median observation period was 56 months (range 3 to 134 months). The median DFS and OS were 46 and 63 months, respectively.

**Figure 2:**
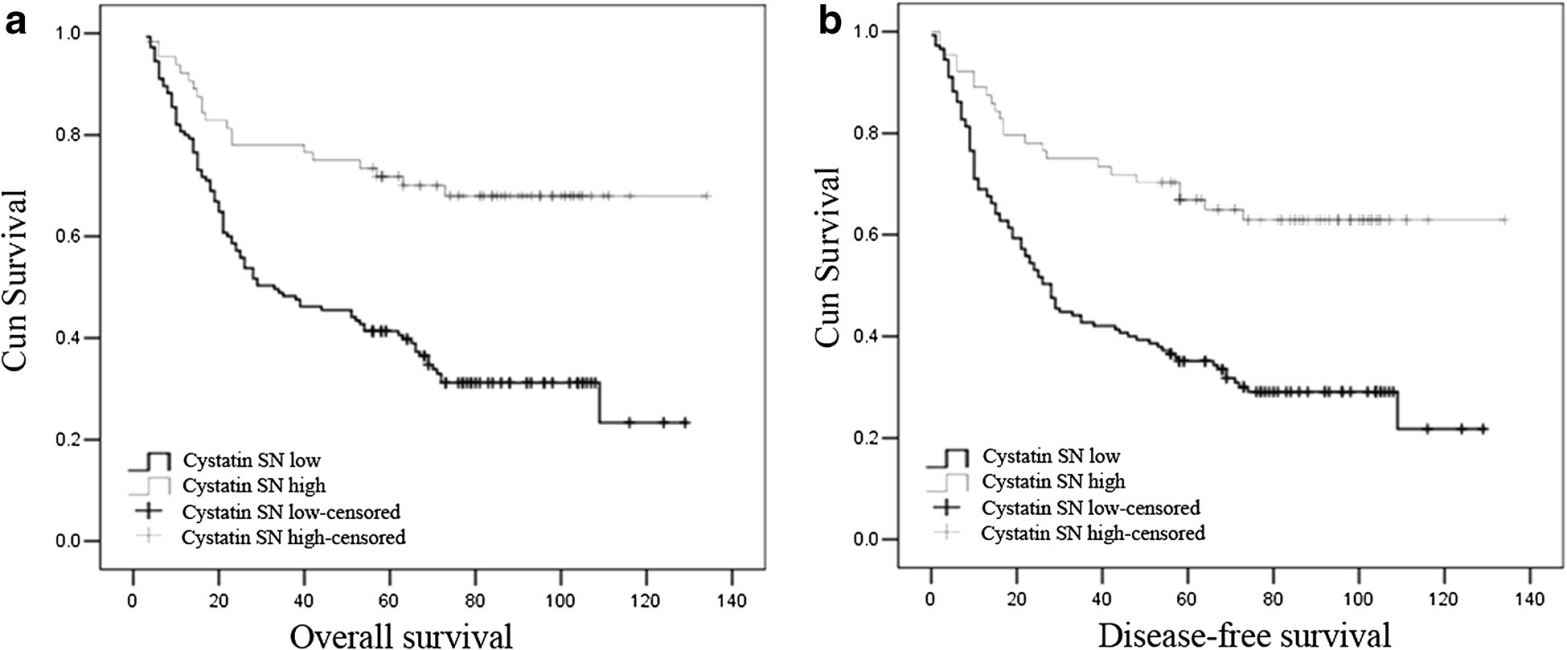
**Kaplan-Meier survival of cystatin SN expression in esophageal squamous cell carcinoma (ESCC) patients.** (**a**) Overall survival and (**b**) disease-free survival curves for the whole cohort of patients with ESCC ( both *P* < 0.001).

In the Kaplan-Meier analysis, the expression of cystatin SN was closely correlated with the DFS and OS of ESCC patients. For the whole cohort, the median DFS and OS were significantly longer for patients with high tumoral expression levels of Cystatin SN than in patients with low tumoural expression levels (both *P* < 0.001, Figure 2, Table 3). Then, we examined the associations between Cystatin SN expression and survival based on the clinicopathological characteristics of the 209 ESCC patients. The results showed that the expression levels of Cystatin SN distinguished the patients with good DFS and OS from patients with poor DFS and OS when the patients were stratified by T status (pT1-2, *P* = 0.001 and *P* = 0.001, respectively; pT3-4, *P* = 0.003 and *P* = 0.001, respectively). This stratification based on the Cystatin SN expression level was also observed for pN0 patients (*P* < 0.001 and *P* < 0.001, respectively), stage II patients (*P* < 0.001 and *P* < 0.001, respectively), tumor grade 1 patients (*P* = 0.003 and *P* = 0.001, respectively) and tumor grade 2 patients (*P* = 0.001 and *P* = 0.002, respectively) (Table 3).

**Table 3:** Prognostic value of Cystatin SN expression in 209 ESCC patients

**Cystatin SN**		**DFS (months)**			**OS (months)**		
**expression**	**Cases**	**Mean**	**Median**	P-value^*^	**Mean**	**Median**	P-value^*^
Total	209			< 0.001			< 0.001
Low expression	145	52	28		57	33	
High expression	64	94	NR		100	NR	
pT status				0.001			0.001
pT1-2	66						
Low expression	43	56	29		60	35	
High expression	23	108	NR		110	NR	
pT3-4	143			0.003			0.001
Low expression	102	49	25		54	26	
High expression	41	73	NR		80	NR	
pN status							
pN0	116			< 0.001			< 0.001
Low expression	76	70	67		75	72	
High expression	40	118	NR		118	NR	
PN1-3	93			0.058			0.017
Low expression	69	29	18		34	21	
High expression	24	44	27		53	42	
pTNM							
Stage II	133			< 0.001			< 0.001
Low expression	88	67	56		72	67	
High expression	45	117	NR		117	NR	
Stage III	76			0.336			0.099
Low expression	57	27	11		31	19	
High expression	19	34	26		45	40	
Differentiation							
G1	56			0.003			0.001
Low expression	39	67	66		71	67	
High expression	17	106	NR		110	NR	
G2	105			0.001			0.002
Low expression	68	49	28		54	35	
High expression	37	92	NR		94	NR	
G3	48			0.604			0328
Low expression	38	37	22		43	25	
High expression	10	49	17		60	23	

^*^Log-rank test.

*DFS* disease-free survival, *OS* overall survival, *pT* pathologic tumor, *pN* pathologic node, *pTNM* pathologic tumor-node-metastasis, *G* tumor grade, *NR* not reached.

A number of factors, including age, gender, tumor location, surgery, differentiation, T status, N status and cystatin SN expression, were used in the univariate Cox regression analysis to assess their impact on the survival of ESCC patients. The variables that were found to impact survival in the univariate analysis were entered into the multivariate analysis model. In this multivariate analysis model, the results showed that significant and independent predictors of survival were the differentiation, N status and Cystatin SN expression (Table 4).

**Table 4:** Univariate and multivariate regression analysis for DFS and OS in the whole cohort

**Variables**	**Disease-free survival**						**Overall survival**					
	**Univariate analysis**			**Multivariate analysis**			**Univariate analysis**			**Multivariate analysis**		
	**HR**	**95% CI**	P value^*^	**HR**	**95% CI**	P value^*^	**HR**	**95% CI**	P value^*^	**HR**	**95% CI**	P value^*^
Age^†^	1.258	0.872-1.813	0.219	.....	.....	.....	1.359	0.933-1.981	0.110	.....	.....	.....
Gender^‡^	0.832	0.535-1.295	0.416	.....	.....	.....	0.759	0.478-1.205	0.242	.....	.....	.....
Location^§^	0.763	0.558-1.043	0.090	.....	.....	.....	0.751	0.542-1.040	0.084	.....	.....	.....
Surgery^¶^	1.034	0.851-1.256	0.738	.....	.....	.....	1.052	0.863-1.284	0.615	.....	.....	.....
pT status^¿^	1.133	0.782-1.644	0.509	.....	.....	.....	1.118	0.772-1.618	0.554	.....	.....	.....
Differentiation^£^	1.393	1.065-1.823	0.016	1.411	1.086-1.833	0.010	1.319	1.004-1.734	0.047	1.331	1.020-1.738	0.035
pN status^ð^	3.211	2.198-4.692	< 0.001	3.096	2.131-4.498	< 0.001	3.032	2.056-4.471	< 0.001	2.926	1.998-4.286	< 0.001
Cystatin SN^ζ^	0.431	0.271-0.685	< 0.001	0.426	0.270-0.672	< 0.001	0.377	0.231-0.616	< 0.001	0.378	0.233-0.614	< 0.001

^*^Cox proportional hazards model; ^†^Age ≤ 57 *vs.* Age > 57; ^‡^Male *vs.* Female; ^§^Upper thoracic *vs.* Middle thoracic *vs.* Lower thoracic; ^¶^Left thoracotomy *vs.* Thoracic-abdominal-cervical incision; ^¿^pT1 *vs.* pT2 *vs.* pT3 *vs.* pT4; ^£^Tumor grade 1 *vs.* Tumor grade 2 *vs.* Tumor grade 3; ^ð^pN0 *vs.* pN1/2/3; ^ζ^High expression of Cystatin SN *vs.* low expression of Cystatin SN. *HR* hazard ratio, *CI* confidence interval, *pT* pathologic tumor, *pN* pathologic node.

## Discussion

The *CST1* gene, which encodes the S-type Cystatin SN peptide, belongs to the Cystatin (CST) superfamily, the members of which control the proteolytic activities of cysteine proteases [5]. Studies have indicated that proteases are involved in both primary and metastatic tumor growth [26,27]. The *CST1* gene is known to play a crucial role in human gastrointestinal tract cancer, including colon cancer and gastric cancer [6,27]. To the best of our knowledge, our study is the first to systematically evaluate the expression and clinicopathologic significance of Cystatin SN in ESCC. Our findings have indicated that Cystatin SN serves as an independent prognostic factor in ESCC patients.

In our study, the immunostaining of ESCC samples revealed that Cystatin SN is predominantly located in the cytoplasm. Previous studies showed that Cystatin SN was present primarily in the cytosolic region of gastric cancer cells, [6] but it was detected in the cytomembrane of colorectal cancer cells [21]. Those observations indicate that the expression of Cystatin SN in different cancers may be tissue–specific [9]. However, we failed to show any significant correlations between Cystatin SN expression and patients clinicopathological parameters. In contrast, some studies revealed that overexpression of Cystatin SN correlated with descending pathological TNM stage for gastric and colorectal cancer. We believe that can explained by the following factors. First, the biology character of Cystatin SN may have tissue specificity. Second, the enrolled ESCCs is only II, III stages and belongs to a single institution’s database, which may results in a selection bias and low statistical power to detect meaningful relationships. Therefore, this concludion merits additional research.

In the studies mentioned above, Cystatin SN, which was proved to contribute to cell proliferation, has been reported as an oncogene in colorectal and gastric caicinoma [6,21]. Howere, in our study, we found that ESCC patients with positive expression of Cystatin SN had significantly longer DFS and OS than those with negative Cystatin SN expression. We believe that can be explained by two particular factors. Firstly, histology differences such as squamous cell carcinoma and adenocarcinoma might explain the observed phenomena. Secondly, the heterogeneity of biomarkers might also result in the discrepancies in the findings between the previous literatures and our study. These results are similar to other members of the CST superfamily in a number of previous reports. Cystatin C is a nonglycosylated 13 kDa basic protein, consisting of 120 amino acids. It belongs to the cystatin superfamily of cysteine proteinase inhibitors. Strojan et al [28] demonstrated significantly longer survival in squamous cell carcinoma of the head and neck patients with high Cystatin C than in those with low Cystatin C. However, in colorectal cance [15], the patients with high levels of Cystatin C exhibited a significantly higher risk of death than those with lower levels. Alterations in secretion may result in higher extracellular and lower intracellular levels of Cystatin C and, therefore, the reverse correlation of Cystatin C with patients’ survival is to be expected. On the other hand, one has to be aware that cysteine proteases and consequently their inhibitors are also involved in biological processes other than tissue remodeling during the progression of primary tumors, such as the regulation of inflammatory and immune responses [29] or apoptosis [30], so that different level of Cystatin C may lead to various clinical outcomes. Moreover, in the subgroup analysis, Cystatin SN expressions distinguish the DFS or OS, especially in the groups of pN0 and stage II patients. Our results suggested that Cystatin SN in ESCC maybe play an significant role in the early stage of carcinogenesis. The underlying mechanism need further study.

The TNM stage is the most powerful and widely accepted predictor of survival for ESCCs [31]. However, many patients with the same stage of disease have different outcomes, indicating the TNM stage may be insufficient to distinguish ESCC patients’ survival [32]. An increasing number of studies have focused on the use of biomarkers to predict patients’ survival and select patients who will benefit from adjuvant treatments. To date, the metastasis and invasiveness of several tumors have been shown to be associated with members of the CST superfamily, such as Cystatin C (encoded by *CST3*), CystatinD (encoded by *CST5*), Cystatin F (encoded by *CST7*), Cystatin M (encoded by *CST6*) and Cystatin S (encoded by *CST4*), which have been described and investigated [11,13-18]. Our study focused on the relationship between one of CST superfamily members and the survival of cancer patients. In our study, Cystatin SN expression, combined with the N status and differentiation, serve as independent and significant predictors in surgically resected ESCCs. Consistent with the findings reported by the previous studies, we also suggested some factors, including age, gender, tumor location, surgery and pT status, were not the independently significant predictive factors for ESCC survival, in spite of some other different points. And some crowd confounding factors may influence the foundings, of course, it needs additional studies. On the other hand, the Cystatin SN expression was shown to distinguish the DFS or OS in a subgroup analysis, especially in the subgroups of pN0 and stage II patients. Therefore, our results indicate that Cystatin SN expression combined with clinicopathological parameters may serve as an extra factor for identifying ESCC patients with a higher risk of tumor recurrence and metastasis.

Unfortunately, one limitation of out study in the lack of confirmation on Cystatin SN expression status by quantitative methods like Reverse Transcription-Polymerase Chain Reaction (RT-PCR), which could, in conjunction with results of IHC, further refine the prognostic value of this biomarker. Also, our study is a retrospective study, relied exclusively on a single-institutional database. Additional mechanistic investigations into this area will be vital to facilitate our understanding of the biological significance of Cystatin SN.

## Conclusions

In conclusion, Cystatin SN expressed higher level in peritumoral normal esophageal mucosae than in the ESCC tissues. Compared with the patients with low expressive level of Cystatin SN, high expression patients have more favourable survivals. Our findings have demonstrated that Cystatin SN expression in ESCC tissue may represent as an independent predictor of survival for patients with resectable ESCC.

## Competing interests

The authors declare that they have no competing interests.

## Authors’ contributions

YFC, GM and XC performed the statistical analysis, drafted the manuscript and participated in the sequence alignment. RZL and LRH participated in the design of the study and participated in the sequence alignment. JHH and MSZ participated in the sequence alignment. ZLH carried out data acquisition. ZSW conceived of the study, and participated in its design and coordination and helped to draft the manuscript. All authors read and approved the final manuscript.

## Pre-publication history

The pre-publication history for this paper can be accessed here:

http://www.biomedcentral.com/1471-2482/13/15/prepub
